# A database of threat statuses and life-history traits of Red List species in Flanders (northern Belgium)

**DOI:** 10.3897/BDJ.7.e34089

**Published:** 2019-04-05

**Authors:** Dirk Maes, Dimitri Brosens, Filiep T’jollyn, Peter Desmet, Frederic Piesschaert, Stijn Van Hoey, Tim Adriaens, Wouter Dekoninck, Koen Devos, Koen Lock, Thierry Onkelinx, Jo Packet, Jeroen Speybroeck, Arno Thomaes, Koen Van Den Berge, Wouter Van Landuyt, Hugo Verreycken

**Affiliations:** 1 Research Institute for Nature and Forest (INBO), Brussels, Belgium Research Institute for Nature and Forest (INBO) Brussels Belgium; 2 Belgian Biodiversity Platform, Brussels, Belgium Belgian Biodiversity Platform Brussels Belgium; 3 Royal Belgian Institute of Natural Sciences, Brussels, Belgium Royal Belgian Institute of Natural Sciences Brussels Belgium; 4 Ugent, Ghent, Belgium Ugent Ghent Belgium; 5 Research Institute for Nature and Forest (INBO), Geraardsbergen, Belgium Research Institute for Nature and Forest (INBO) Geraardsbergen Belgium

**Keywords:** Red List, Flanders (northern Belgium), life-history traits, IUCN, threatened species, conservation

## Abstract

**Background:**

Red Lists estimate the extinction risk of species at global or regional levels and are important instruments in conservation policies. Global Red List assessments are readily available via the IUCN website (https://www.iucnredlist.org) and are regularly updated by (taxonomic) experts. Regional Red Lists, however, are not always easy to find and often use local criteria to assess the local extinction risk of species.

**New information:**

Here, we publish a database with the outcome of 38 Red List assessments in Flanders (northern Belgium) between 1994 and 2018. In total, the database contains 6,224 records of 5,039 unique taxa pertaining to 24 different taxonomic groups. Using a quality control procedure, we evaluated the criteria used, the number of records, the temporal and spatial distribution of the data and the up-to-dateness of the Red Lists. This way, nineteen Red Lists were approved as being of sufficient high quality (i.e. validated) and nineteen others were not. Once validated, Red Lists are approved by the regional Minister of Environment and published in the Belgian Official Gazette acquiring legal status. For the validated Red Lists, we additionally compiled (life-history) traits that are applicable to a wide variety of species groups (taxonomic kingdom, environment, biotope, nutrient level, dispersal capacity, lifespan and cuddliness). The publication of this dataset allows comparison of Red List statuses with other European regions and countries and permits analyses about how certain (life-history) traits can explain the Red List status of species. The dataset will be regularly updated by adding new Red List (re)assessments and/or additional (life-history) traits.

## Introduction

Red Lists are important instruments at both the global and the regional scale (Brooks et al. 2016). They estimate the extinction risk in a given region, usually using standardised and internationally accepted criteria (Mace et al. 2008). Although Red Lists are not compiled to prioritise conservation actions (Lamoreux et al. 2003, Rodrigues et al. 2006), they are often used as an important source for conservation policies (McCarthy et al. 2008), such as species action plans (Fitzpatrick et al. 2007, Laycock et al. 2011) and reintroduction programmes (IUCN/SSC 2013). Additionally, consecutive Red Lists allow comparison of changes in Red List categories and, in combination with information on threats, provide information about effective application of conservation efforts to the species’ major threats (Brooke et al. 2008). Yet, national or regional Red Lists are often only available locally and/or in the local language and not always easy to access, which hinders analyses on larger scales (Maes et al. 2019). Although a website with national or regional Red Lists exists (http://www.nationalredlist.org), it is often more convenient for local authorities to manage the information on national or regional Red Lists locally (e.g. https://www.inbo.be/en/search-flanders-red-lists) and to publish them on open-access platforms for easier availability.

Here, we publish the results of Red List assessments in Flanders (northern Belgium) between 1994 and 2018. For the species on the validated Red Lists, we also include some general (life-history) traits (taxonomic kingdom, environment, biotope use, nutrient level, lifespan, mobility and cuddliness - cf. Trochet et al. 2014), which allows further analyses on the correlation between the Red List status and the species characteristics (cf. Jeppsson and Forslund 2014).

## General description

### Purpose

This database publishes the Red List statuses of all species that were assessed in Flanders (northern Belgium) since 1994. First, a literature search was done in both local and scientific publications to gather all Red List assessments ever performed in Flanders. All species present in the Red Lists were compiled in a database with the original taxonomic name and Red List status as published in the original Red List. The data were carefully checked for double entries and for typing errors in the published species names. Since Red List categories were not always in accordance with the presently-used IUCN categories, we ''translated'' the originally-published Red List category into IUCN Red List categories. Second, all species names were checked against the GBIF Backbone Taxonomy (GBIF secretariat 2019) to obtain currently traceable species names (including synonyms). Since the Flemish Species Decree of 2009 (Flemish Government 2009) came into effect, Red List assessments in Flanders are coordinated by the Research Institute for Nature and Forest (INBO, formerly the Institute for Nature Conservation – IN). The task of the Institute is to not only instigate the compilation of new Red Lists, but also to perform a control procedure to check whether the Red List is of sufficiently high quality. The quality control procedure consists of checking the number of available historical and recent data, the number of sites that were surveyed in both historical and recent times (>70 grid cells of 5 x 5 km) and a minimal spatial coverage (>10%) of the different ecological districts of Flanders (Maes et al. 2015). For older Red Lists, this quality control was done *post factum*, while for new Red Lists, this was done at the start of the Red List assessment. Red Lists fulfilling the quality control criteria are labelled as ''validated Red Lists''. Applying this procedure, we were able to validate nineteen out of 38 published Red Lists Table 1, while nineteen others could not be validated Table 2.

Thirteen validated Red Lists have already been approved by the Minister of Environment, were published in the Belgian Official Gazette and thus acquired legal status (publication numbers 2011035522 and 2013204362; http://www.ejustice.just.fgov.be/doc/rech_n.htm). The procedure to have the six most recently validated Red Lists also approved by the Minister has been started (saproxylic beetles, breeding birds, grasshoppers, hornworts, liverworts and mosses – Table 1).

**Figure 1.**
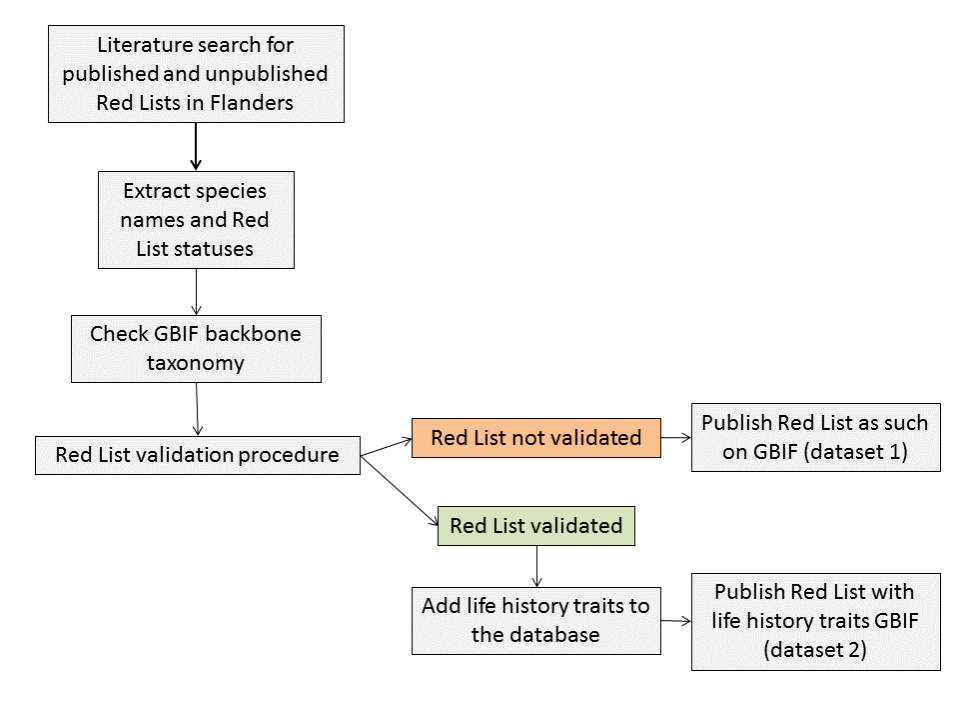
Workflow for the compilation of the Red List database in Flanders (northern Belgium).

Finally, we added (life-history) traits (kingdom, environment, biotope, nutrient level, lifespan, mobility and cuddliness) to the species in the validated Red Lists based on regional sources on the biology and/or ecology of the different species groups (see references in Table 1). These life-history traits are explained in Tables 5, 6, 7, 8, 9, 10, 11. The workflow for the compilation of the Red List database in Flanders is given in Fig. 1.

We will update the database regularly, i.e. whenever new Red Lists are published and/or new information on the life-history traits of the assessed species becomes available. We also aim to reassess all Red Lists (using IUCN criteria) of taxonomic groups for which only local criteria were applied in the past (e.g. carabid beetles, dragonflies, vascular plants).

### Additional information

Red Lists are usually published as reports from the Research Institute for Nature and Forest (INBO), but some of them remained unpublished and were only available for internal use (waterbeetles – Bosmans 1994, waterbugs – Bosmans 1994; Bonte et al. 2001, hoverflies – Meerhaeghe and Grootaert 1998, land snails – van Loen et al. 2006). Other Red Lists were published as part of (provisional) distribution atlases (amphibians and reptiles – Bauwens and Claus 1996, freshwater fishes – Vandelannoote and Coeck 1998, grasshoppers – Decleer et al. 2000, ants – Dekoninck et al. 2003, breeding birds – Devos et al. 2004, dragonflies – De Knijf 2006, vascular plants – Van Landuyt et al. 2006), as dissertation projects (waterbeetles – Scheers 2012) or as papers in local (spiders – Maelfait et al. 1998, waterbugs – Lock et al. 2013) or international journals (butterflies – Maes et al. 2012, freshwater fishes – Verreycken et al. 2014, ladybirds - Adriaens et al. 2015). In this database, we bring together all the threat statuses of all the species mentioned in the published and unpublished Red Lists since 1994 in Flanders.

Since 1994, 38 Red Lists have been compiled in Flanders. In total, this concerned 6,224 records of 5,039 unique species – in some cases listed in consecutive Red Lists - pertaining to 24 taxonomic groups. Most of the older Red Lists (1994-1999) used expert judgement without quantitative analyses to classify species into Red List categories: mammals (Criel 1994), waterbugs (Bosmans 1994), waterbeetles (Bosmans 1994), spiders (Maelfait et al. 1998) and breeding birds (Devos and Anselin 1999). After the publication of local Red List criteria (Maes et al. 1995), Red Lists were compiled using a combination of rarity and decline (Maes and van Swaay 1997). Since 2011, however, IUCN criteria for regional use (IUCN 2003) were adopted in Flanders (Maes et al. 2011) and since then, all Red Lists were compiled using the IUCN criteria. The main difference between local and IUCN criteria is that IUCN criteria allow species to be classified based on declining trends (criterion A), rarity or small population sizes only (criterion B, C or D), while in the previously used local criteria, a species could only be classified as threatened when it had both a declining (distribution or population) trend and when the species had a limited distribution.

## Geographic coverage

### Description

**Figure 2.**
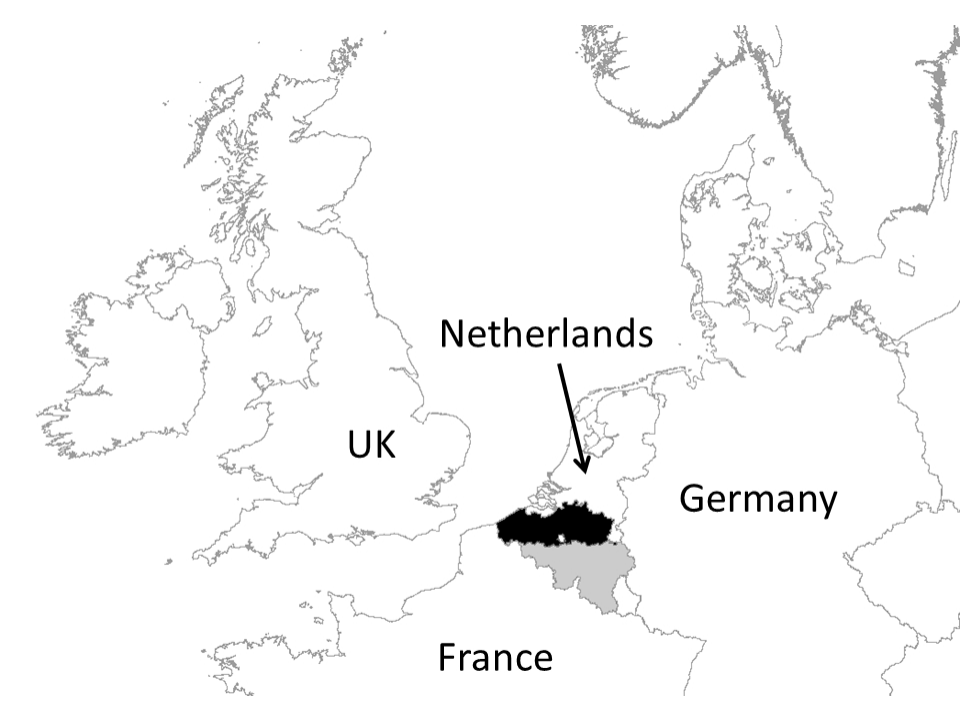
The location of Flanders (in black) within Belgium (in grey) in NW Europe. The white area within Flanders is the Brussels-Capital Region.

Flanders covers an area of 13,522 km² and is the northern administrative region of Belgium Fig. 2. The bounding box of Flanders is 50.67N to 51.51N latitude and 2.53E to 5.94E longitude. It represents 45% of Belgian territory and is largely covered by agricultural land and urban areas. Only 15% of Flemish territory is covered by (semi-)natural areas (e.g. woodlands, wetlands, heathlands, grasslands). With 481 inhabitants per km², Flanders is one of the most densely populated areas in Europe.

### Coordinates

50.67 and 51.51 Latitude; 5.94 and 2.53 Longitude.

## Traits coverage

**RLCAsPublished and RLC_IUCN**

The Red List category as published (RLCAsPublished) in the original Red Lists, mostly in Dutch, are given in Table 3, as well as their translation into the Red List category (RLC_IUCN) according to the IUCN Red List categories (IUCN 2003). Depending on the Red List categories used, the original Red List categories can be translated into different IUCN categories and vice versa.

**Criteria**

The criteria used to compile the Red Lists (Table 4).

**Kingdom**

The taxonomic kingdom to which a species belongs (Fungi, Invertebrates, Plants, Vertebrates – Table 5).

**Environment**

The environment in which the species occurs (Aquatic, Epiphytic, Marine, Semi-aquatic, Terrestrial – Table 6).

**Biotope**

The preferred biotope in which the species occurs in Flanders (northern Belgium) (Table 7). This is based on broad biotope classifications used in the land use map of Flanders (Gobin et al. 2009) and in Corine Land Cover (Version 18.5.1) or Natura2000 habitats (Council Directive 92/43/EEC). For species occurring in two different biotope types, both biotopes are given in the dataset.

**NutrientLevel**

The nutrient level of the biotope in which the species occurs (Eutrophic, Mesotrophic, Oligotrophic – Table 8).

**Lifespan**

The longevity of the species (Table 9). We arbitrarily choose 3 years to discriminate between longlived and shortlived species.

**Mobility**

The dispersal capacities of the species (Table 10) We arbitrarily choose 5 kilometres to discriminate between mobile and sedentary species.

**Cuddliness**

Whether the species is considered cuddly or not (Stokes 2007) (Table 11). This information is only given for animals (invertebrates and vertebrates).

## Temporal coverage

### Notes

All Flemish Red Lists compiled between 1994 and 2018.

## Usage rights

### Use license

Creative Commons Public Domain Waiver (CC-Zero)

## Data resources

### Data package title

Validated & non-validated Red Lists of Flanders, Belgium

### Number of data sets

2

### Data set 1.

#### Data set name

Non-validated Red List of Flanders, Belgium

#### Data format

DwC-A

#### Number of columns

2

#### Character set

UTF-8

#### Download URL

https://doi.org/10.15468/54nwog

#### Description

The Non-validated Red Lists of Flanders, Belgium is a species checklist dataset published by the Research Institute for Nature and Forest (INBO). It includes 3,161 taxa from 19 Flemish Red Lists that are considered non-validated, i.e. which did not use quantitative criteria and a representative sample of occurrences across all ecological regions in Flanders (Maes et al. 2015) for Red List assessment. Here, this compilation is published as a standardised Darwin Core Archive and includes for each taxon: the scientific name, higher classification (provided by the GBIF Backbone Taxonomy, https://doi.org/10.15468/39omei), stable taxon identifier and Dutch vernacular name (in the taxon core) and the Red List category in Flanders as published, its IUCN equivalent and year of assessment (respectively, in occurrenceRemarks, threatStatus and eventDate in the distribution extension). Issues with the dataset can be reported at: https://github.com/inbo/rl-flanders-checklist.

**Data set 1.** 

Column label	Column description
Taxon core	http://rs.tdwg.org/dwc/terms/Taxon
Distribution extension	http://rs.gbif.org/terms/1.0/Distribution

### Data set 2.

#### Data set name

Validated Red Lists of Flanders, Belgium

#### Data format

DwC-A

#### Number of columns

3

#### Character set

UTF-8

#### Download URL

https://doi.org/10.15468/8tk3tk

#### Description

The Validated Red Lists of Flanders, Belgium is a species checklist dataset published by the Research Institute for Nature and Forest (INBO). It includes 3,063 taxa from 19 Flemish Red Lists that are considered validated, i.e. which used quantitative criteria and a representative sample of occurrences across all ecological regions in Flanders (Maes et al. 2015) for Red List assessment. Here, this compilation is published as a standardised Darwin Core Archive and includes for each taxon: the scientific name, higher classification (provided by the GBIF Backbone Taxonomy, https://doi.org/10.15468/39omei), stable taxon identifier and Dutch vernacular name (in the taxon core), the Red List category in Flanders as published, its IUCN equivalent and year of assessment (respectively, in occurrenceRemarks, threatStatus and eventDate in the distribution extension) and the life-history traits environment, biotope, cuddliness, lifespan, mobility, nutrient level and spine (in the description extension). Issues with the dataset can be reported at: https://github.com/inbo/rl-flanders-checklist.

**Data set 2.** 

Column label	Column description
Taxon core	http://rs.tdwg.org/dwc/terms/Taxon
Distribution extension	http://rs.gbif.org/terms/1.0/Distribution
Description extension	http://rs.gbif.org/extension/gbif/1.0/description.xml

## Figures and Tables

**Table 1.** Validated Red Lists in Flanders (n = 19) with the Red List criteria used (Local or IUCN Red List criteria), the year of publication, the reference to the Red List, the reference to the life-history traits and the number of species (nSpecies) included in the Red List. Red Lists marked with a * have been approved by the minister.

**Taxonomic group**	**Criteria**	**Year**	**Reference**	**Reference(s) life-history traits**	**nSpecies**
Butterflies (Lepidoptera – Rhopalocera)*	Local	1996	Maes and Van Dyck (2001)	Maes et al. (2013)	68
Grasshoppers (Orthoptera)*	Local	2000	Decleer et al. (2000)	Kleukers et al. (1997)	39
Breeding birds (Aves)*	Local	2004	Devos et al. (2004)	Birdlife International (2004), Vermeersch et al. (2004)	211
Dragonflies (Odonata)*	Local	2005	De Knijf (2006)	De Knijf (2006), Nederlandse vereniging voor Libellenstudie (2002)	66
Vascular plants (Tracheophyta)*	Local	2006	Van Landuyt et al. (2006)	Biesbrouck et al. (2001), Ellenberg et al. (1992), Hill et al. (2004), Stieperaere and Fransen (1982)	1154
Carabid beetles (Coleoptera – Carabidae)*	Local	2008	Desender et al. (2008b)	Desender et al. (2008a), Homburg et al. (2014), Turin (2000)	382
Butterflies (Lepidoptera – Rhopalocera)*	IUCN	2011	Maes et al. (2012)	Maes et al. (2013)	70
Amphibians (Amphibia)*	IUCN	2012	Jooris et al. (2012)	Jooris et al. (2013)	16
Reptiles (Reptilia)*	IUCN	2012	Jooris et al. (2012)	Jooris et al. (2013)	6
Waterbugs (Hemiptera – Gerromorpha, Nepomorpha)*	IUCN	2013	Lock et al. (2013)	Aukema et al. (2002), Stoffelen et al. (2013)	62
Freshwater fishes (Pisces)*	IUCN	2014	Verreycken et al. (2014)	Kottelat and Freyhof (2007), Vandelannoote et al. (1998), Van Emmerik and De Nie (2006)	42
Ladybirds (Coleoptera – Coccinellidae)*	IUCN	2014	Adriaens et al. (2015)	Adriaens and Maes (2004), Adriaens et al. (2008), Baugnée et al. (2011), Cuppen et al. (2017), Roy et al. (2011)	39
Mammals (Mammalia)*	IUCN	2014	Maes et al. (2014)	Verkem et al. (2003)	103
Saproxylic beetles (Coleoptera – Cetoniidae, Dynastidae, Lucanidae)	IUCN	2015	Thomaes et al. (2015)	Thomaes et al. (2015)	19
Breeding birds (Aves)	IUCN	2016	Devos et al. (2016)	Birdlife International (2004), Vermeersch et al. (2004)	217
Grasshoppers (Orthoptera)	IUCN	2017	Maes et al. (2017)	Kleukers et al. (1997)	52
Hornworts (Anthocerotophyta)	IUCN	2017	Van Landuyt and De Beer (2017)	Hill et al. (2007), Siebel (2005) , Siebel and During (2006)	4
Liverworts (Marchantiophyta)	IUCN	2017	Van Landuyt and De Beer (2017)	Hill et al. (2007), Siebel (2005), Siebel and During (2006)	114
Mosses (Bryophyta)	IUCN	2017	Van Landuyt and De Beer (2017)	Hill et al. (2007), Siebel (2005), Siebel and During (2006)	399

**Table 2.** Non-validated Red Lists in Flanders (n = 19) with the criteria used (Expert judgement, Local or IUCN Red List criteria), the year of publication, the reference to the Red List and the number of species (nSpecies) included in the Red List.

**Taxonomic group**	**Criteria**	**Year**	**Reference**	**nSpecies**
Mammals (Mammalia)	Expert judgement	1994	Criel (1994)	69
Waterbugs (Hemiptera – Gerromorpha, Nepomorpha)	Expert judgement	1994	Bosmans (1994)	58
Waterbeetles (Coleoptera – Dytiscidae, Gyrinidae, Haliplidae, Noteridae, Paelobiidae)	Expert judgement	1994	Bosmans (1994)	139
Carabid beetles (Coleoptera – Carabidae)	Local	1995	Desender et al. (1995)	368
Amphibians (Amphibia)	Local	1996	Bauwens and Claus (1996)	14
Dragonflies (Odonata)	Local	1996	De Knijf and Anselin (1996)	58
Reptiles (Reptilia)	Local	1996	Bauwens and Claus (1996)	5
Fishes (Pisces)	Local	1998	Vandelannoote and Coeck (1998)	55
Hoverflies (Diptera – Syrphidae)	Local	1998	Meerhaeghe and Grootaert (1998)	265
Spiders (Araneae)	Expert judgement	1998	Maelfait et al. (1998)	604
Breeding birds (Aves)	Expert judgement	1999	Devos and Anselin (1999)	71
Macrofungi (Ascomycota, Basidiomycota)	Local	1999	Walleyn and Verbeken (1999)	552
Dolichopodid flies (Diptera – Dolichopodidae)	Local	2000	Pollet (2000)	260
Empidid flies (Diptera – Empididae, Hybotidae, Atelestidae, Brachystomatidae)	Local	2001	Grootaert et al. (2001)	259
Waterbugs (Hemiptera – Gerromorpha, Nepomorpha)	Local	2001	Bonte et al. (2001)	58
Ants (Hymenoptera – Formicidae)	Local	2003	Dekoninck et al. (2003)	55
Land snails (Mollusca)	Local	2006	van Loen et al. (2006)	120
Waterbeetles (Coleoptera – Dytiscidae)	Local	2012	Scheers (2012)	106
Grasshoppers (Orthoptera)	IUCN	2013	Lock et al. (2011)	45

**Table 3.** Translation of published Red List category name into IUCN Red List categories.

**RLCAsPublished**	**RLC_IUCN**	**IUCN Category**
Achteruitgaand	NT	Near Threatened
Bedreigd	EN	Endangered
Bedreigd	VU	Vulnerable^a^
Bedreigd, maar mate waarin ongekend	DD	Data Deficient
Bedreigd, maar niet gekend in welke mate	DD	Data Deficient
Bijna in gevaar	NT	Near Threatened
Critically endangered	CR	Critically Endangered
Endangered	EN	Endangered
Ernstig bedreigd	CR	Critically Endangered
Geografisch beperkt	NT	Near Threatened
Kwetsbaar	VU	Vulnerable
Least concern	LC	Least Concern
Met uitsterven bedreigd	CR	Critically Endangered
Met verdwijning bedreigd	CR	Critically Endangered
Momenteel niet bedreigd	LC	Least Concern
Momenteel niet in gevaar	LC	Least Concern
Near threatened	NT	Near Threatened
Niet bedreigd	LC	Least Concern
Niet geëvalueerd	NE	Not Evaluated
Niet van toepassing	NA	Not Applicable
Niet-inheemse broedvogel	NA	Not Applicable
Not assessed	NE	Not Evaluated
Onregelmatige broedvogel	NE	Not Evaluated
Onvoldoende data	DD	Data Deficient
Onvoldoende gekend	DD	Data Deficient
Regionaal uitgestorven	RE	Regionally Extinct
Regionally extinct	RE	Regionally Extinct
Sterk bedreigd	EN	Endangered
Uitgestorven	RE	Regionally Extinct
Uitgestorven in Vlaanderen	RE	Regionally Extinct
Vatbaar voor bedreiging	NT	Near Threatened
Verdwenen	RE	Regionally Extinct
Verdwenen uit Vlaanderen en het Brussels Gewest	RE	Regionally Extinct
Vermoedelijk bedreigd	DD	Data Deficient
Vulnerable	VU	Vulnerable
Waarschijnlijk bedreigd	DD	Data Deficient
Zeldzaam	NT	Near Threatened
Zeldzaam (vrij zeldzaam)	NT	Near Threatened
Zeldzaam (zeer zeldzaam)	NT	Near Threatened
Zeldzaam (zeldzaam)	NT	Near Threatened

**Table 4.** Criteria used for the Red List assessments in Flanders.

**Criteria**	**Description**
Expert judgement	The Red List was compiled on the basis of expert knowledge about rarity and trend without the use of quantitative criteria
Local	Quantitative local criteria were used (Maes et al. 1995)
IUCN	Quantitative IUCN criteria were used (Maes et al. 2011)

**Table 5.** The taxonomic kingdom to which the different species groups belong.

**Kingdom**	**Description**
Fungi	Agaricomycetes, Geoglossomycetes, Leotiomycetes, Pezizomycetes, Sordariomycetes
Invertebrates	Ants (Formicidae), Butterflies (Lepidoptera – Rhopalocera), Carabid beetles (Carabidae), Dolichopodid flies (Dolichopodidae), Dragonflies (Odonata), Empidid flies (Empididae, Hybotidae, Atelestidae, Brachystomatidae), Grasshoppers (Orthoptera), Hoverflies (Diptera – Syrphidae), Ladybirds (Coleoptera – Coccinellidae), Molluscs (Mollusca), Saproxylic beetles (Coleoptera – Cetoniidae, Dynastidae, Lucanidae), Spiders (Araneae), Waterbeetles (Coleoptera - Dytiscidae, Gyrinidae, Haliplidae, Noteridae, Paelobiidae), Waterbugs (Hemiptera – Gerromorpha, Nepomorpha)
Plants	Hornworts (Anthocerotophyta), Liverworts (Marchantiophyta), Mosses (Bryophyta), Vascular plants (Tracheophyta)
Vertebrates	Amphibians (Amphibia), Breeding birds (Aves), Freshwater fishes (Pisces), Mammals (Mammalia), Reptilia (Reptilia)

**Table 6.** Description of the environment in which each species occurs.

**Environment**	**Description**
Aquatic	The major part of the life cycle is in water
Epiphytic	Living on trees (only liverworts and mosses)
Marine	At least a part of the life cycle is in the sea
Semi-aquatic	Water is necessary for hunting or breeding
Terrestrial	The major part of the life cycle is on land

**Table 7.** The biotope type in which each species occurs.

**Biotope**	**Description**
Agriculture	Arable fields, agricultural grasslands
Dunes	Sandy shores and coastal dunes
Eurytopic	No clear biotope preference or occurring in different biotope types
Grasslands	Dry and wet semi-natural grasslands
Heathlands	Dry and wet heathlands, (peat)bogs
Marine	Sea
Marshes	Wetlands, mires
Running waters	Rivers, rivulets
Salt marshes	Littoral sediment
Shrubs	Scrubs
Standing waters	Ponds, lakes, ditches
Urban	Anthropogenic (buildings, gardens, cemeteries, railroads …), industrial sites
Woodlands	Deciduous, coniferous and mixed woodlands

**Table 8.** The nutrient level of the biotope in which each species occurs.

**Nutrient level**	**Description**
Eutrophic	The biotope in which the species occurs has a high nutrient level
Mesotrophic	The biotope in which the species occurs has an intermediate nutrient level
Oligotrophic	The biotope in which the species occurs has a low nutrient level

**Table 9.** The lifespan of each species.

**Lifespan**	**Description**
Longlived	The species lives ≥ 3 years
Shortlived	The species lives < 3 years

**Table 10.** The dispersal capacity of each species.

**Mobility**	**Description**
Mobile	The dispersal capacity of the species is ≥ 5 km
Sedentary	The dispersal capacity of the species is < 5 km

**Table 11.** The cuddliness of each species.

**Cuddliness**	**Description**
Cuddly	The species is considered cuddly
Non cuddly	The species is considered non-cuddly (spiny, dangerous, venomous, predator)
